# Mental health impact of intragroup vs. intergroup wartime violence

**DOI:** 10.1093/pnasnexus/pgag058

**Published:** 2026-03-31

**Authors:** Joan Barceló, Keshana Ratnasingham

**Affiliations:** Social Science Division, New York University Abu Dhabi, Saadiyat Island, Abu Dhabi 129188, United Arab Emirates; Social Science Division, New York University Abu Dhabi, Saadiyat Island, Abu Dhabi 129188, United Arab Emirates

**Keywords:** wartime violence, PTSD, posttraumatic growth, Sri Lanka, civil war

## Abstract

Exposure to political violence has well-documented consequences for psychological well-being. Yet, most research emphasizes the consequences of intergroup conflict and overlooks that of intragroup violence. Using survey data from Tamil civilians in postwar Sri Lanka, we compare the mental health outcomes of those exposed to violence perpetrated by ethnic outgroups (intergroup violence) and those exposed to violence by members of their own ethnic group (intragroup violence). Results show that both forms of violence increase posttraumatic stress disorder, but the impact of intragroup violence is nearly four times larger. In contrast, intergroup violence is positively associated with posttraumatic growth), while intragroup violence is not. These findings highlight the need to consider the source of violence in designing postconflict mental health interventions.

## Introduction

Political violence leaves deep psychological scars, with long-term impacts on individuals’ mental health, social cohesion, and postconflict recovery (1–3). In recent decades, extensive cross-national research has documented elevated rates of trauma-related disorders, particularly posttraumatic stress disorder (PTSD) and depression, among populations affected by armed conflict (4–6). These studies span a wide range of conflict zones, including Afghanistan, Iraq, Uganda, Syria, and the Balkans, highlighting both the scale and durability of wartime psychological harm (7, 8).

Much of this literature focuses on violence perpetrated between opposing identity groups—typically defined along ethnic, religious, or national lines—and emphasizes the role of intergroup trauma in shaping outcomes like PTSD, depression, or prejudice (5, 6, 9). This focus is grounded in evidence that intergroup violence can entrench social divisions, increase outgroup hostility, and disrupt trust across ethnic lines (10). However, such approaches often neglect a critical dimension of many civil conflicts: intragroup violence, or violence perpetrated by coethnics and fellow combatants (11).

Intragroup violence is common in civil wars. Approximately 45% of conflicts since the Cold War have involved significant levels of infighting within rebel movements or civilian communities (11). Such violence may be particularly destabilizing, as it threatens victims’ sense of identity, trust in social networks, and access to traditional sources of psychological support. In contrast to intergroup violence, which may galvanize collective solidarity, intragroup violence can trigger betrayal, cognitive dissonance, and social isolation (12).

Despite its prevalence, few studies have systematically examined the psychological consequences of intragroup violence, particularly in comparison to intergroup violence. Existing evidence suggests that the identity of the perpetrator can shape trauma outcomes by influencing both perceived threat and access to coping resources (12). This study helps fill that gap by directly comparing the long-term mental health effects of exposure to intragroup and intergroup violence.

We draw on original survey data collected in 2022 from 628 Tamil adults in Jaffna district, Northern Sri Lanka, a region deeply affected by the country’s 26-year civil war. The Sri Lankan conflict featured both interethnic violence and internal repression by the Liberation Tigers of Tamil Eelam, the main Tamil rebel group. This dual context allows us to compare how exposure to intergroup violence (by Sinhalese forces) and intragroup violence (by Tamil actors) independently shape mental health outcomes years after the war.

We examine two psychological outcomes, PTSD and posttraumatic growth (PTG). PTSD captures adverse psychological responses to trauma exposure, whereas PTG reflects positive changes such as greater appreciation for life or strengthened personal resilience (13). Prior work studies these outcomes in postwar populations, but we contribute by directly assessing whether the identity of the perpetrator matters.

The identity of the perpetrator has important theoretical and policy implications. Intragroup violence is especially harmful because it erodes trust within one’s community, disrupts support networks, and generates betrayal and identity dissonance. By contrast, intergroup violence can be more easily processed through external blame, which may buffer PTSD and, in some cases, foster PTG by providing clearer narratives that aid coping. In short, intragroup violence is likely to heighten PTSD and limit PTG, whereas intergroup violence may still allow pathways to growth. Guided by this reasoning, we hypothesize that intergroup violence will be associated with higher PTSD and PTG, while intragroup violence will produce greater increases in PTSD and offer limited scope for PTG.

## Results and discussion

**Fig. 1.**
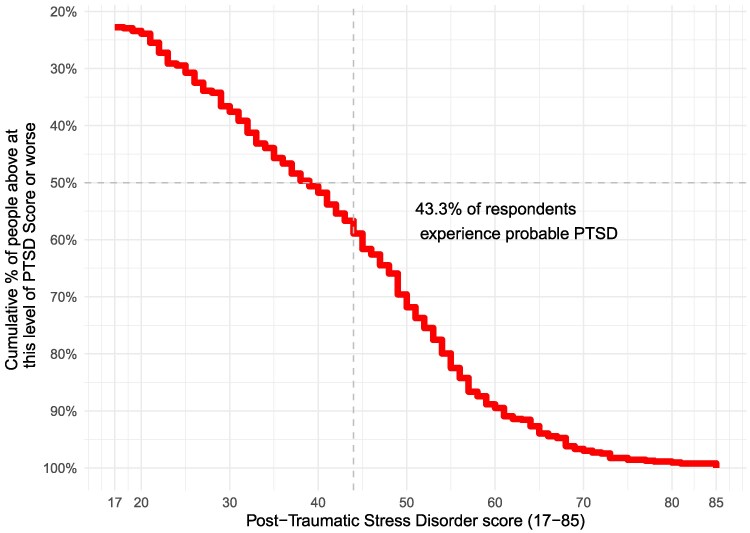
Prevalence of PTSD in contemporary Jaffna.

**Fig. 2.**
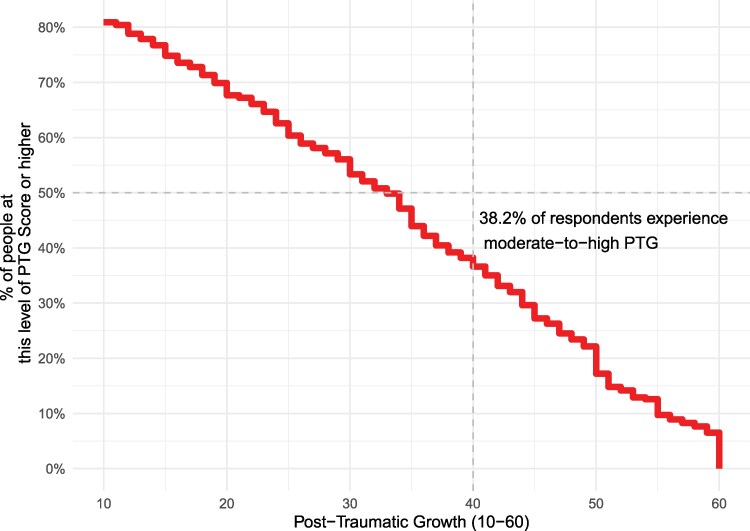
Prevalence of PTG in contemporary Jaffna.

Severe PTSD prevalence in contemporary Jaffna is 43.3% (95% CI 39.5–47.3), using a PCL-C threshold of 44 or higher (Fig. 1). At the same time, 38.2% of residents show moderate-to-high PTG (95% CI 34.4–42.0), using a PTG-SF threshold of 40 or higher (Fig. 2). The coexistence of high PTSD and PTG underscores the multidimensional nature of trauma recovery, where individuals may struggle with distress while also finding ways to grow.

Table 1 shows significant differences by perpetrator type. Each additional traumatic event related to Sinhalese (intergroup) violence raised PTSD scores by 0.09--0.12 (P<0.01), equivalent to about one unit for every 10 events. In contrast, Tamil (intragroup) violence had a much stronger effect, increasing PTSD scores by 0.36–0.43 per event (P<0.01), or about 3.6–4.3 units per 10 events. On a 0–140 scale, this indicates that intragroup violence inflicts three to five times more trauma than intergroup violence, a difference that persists 13 years after the war.

**Table 1.** The effect of exposure to Sinhalese and Tamil violence on mental health outcomes

	PTSD score			PTG score		
	(1)	(2)	(3)	(4)	(5)	(6)
Exposure to Sinhalese	0.11${\;}^{a}$	0.12${\;}^{a}$	0.09${\;}^{a}$	0.13${\;}^{a}$	0.13${\;}^{a}$	0.10${\;}^{a}$
(intergroup violence)	(0.03)	(0.03)	(0.03)	(0.03)	(0.03)	(0.03)
Exposure to Tamil	0.36${\;}^{a}$	0.43${\;}^{a}$	0.39${\;}^{a}$	− 0.02	− 0.02	− 0.03
(intragroup violence)	(0.10)	(0.10)	(0.10)	(0.10)	(0.10)	(0.10)
Female			− 4.34${\;}^{a}$			− 1.56
			(1.34)			(1.38)
Age			0.06			0.16${\;}^{a}$
			(0.04)			(0.05)
Education			− 0.55			0.43
			(0.59)			(0.61)
Father’s education			− 0.19			− 0.04
			(0.59)			(0.61)
Mother’s education			− 1.03			0.56
			(0.66)			(0.68)
Family ownership			− 0.70			− 0.18
			(1.31)			(1.35)
Family wealth			− 0.93			− 1.45${\;}^{c}$
			(0.76)			(0.78)
GN fixed effects	N	Y	Y	N	Y	Y
Observations	628	628	628	628	628	628

Significance levels: ^a^*P* < 0.01; ^b^*P* < 0.05; ^c^*P* < 0.1.

Turning to PTG, exposure to Sinhalese violence was positively associated with growth (0.10–0.13 per event, P<0.01), while Tamil violence showed no such effect. This pattern suggests that individuals may reframe trauma and derive personal growth when it can be attributed to an identifiable outgroup, but intragroup violence disrupts this process by provoking identity dissonance, inhibiting the psychological processes typically associated with PTG.

Although intragroup and intergroup exposures are only weakly correlated (r=0.15), about 11% of respondents experienced above-mean exposure to both. Appendix S1 reports models that compare individuals with high exposure to one type of violence against those with high exposure to both. The findings show that PTSD is highest among respondents exposed only to intragroup violence, and although exposure only to intergroup violence is also associated with higher PTSD, that effect is significantly smaller. When both experiences are present, PTSD does not worsen further, rather, the presence of intergroup violence appears to mitigate the negative effect of intragroup violence, making the intragroup component seemingly less relevant, which is consistent with the idea that intergroup exposure can enable blame shifting to an external perpetrator and thereby buffer trauma. For PTG, intergroup violence remains the main driver: growth is strongest among those primarily exposed to Sinhalese violence, and among dual-exposure individuals the intergroup component dominates, allowing PTG to emerge despite concurrent intragroup violence.

Overall, intragroup violence imposes a heavier psychological burden, reflected in higher PTSD, whereas posttraumatic growth tends to follow intergroup violence and is largely absent after intragroup violence. These patterns hold at comparable exposure levels, which indicates that the source of harm, not only its severity, matters. A plausible mechanism is betrayal and role conflict within one’s own community, which can amplify fear, mistrust, and moral injury, thereby impeding meaning making and recovery. By contrast, intergroup violence, although traumatic, often permits clearer attribution to an external perpetrator, which can support narrative coherence and, for some, activate pathways to psychological growth. These results align with our hypothesis and with calls to disaggregate the legacies of violence by perpetrator identity. For practice and policy, screening and treatment should be adapted to both exposure severity and perpetrator identity, with intragroup cases prioritizing community reintegration.

While these findings are robust across specifications, limitations should be noted. The cross-sectional design limits causal inference, and our reliance on self-reported measures raises the possibility of recall bias. Future research should extend this analysis to other postconflict societies, employ longitudinal data to track how PTSD and PTG evolve over time, and more systematically examine how individual-level factors such as gender or age at exposure may condition the mental health outcomes of different forms of wartime violence.

## Materials and methods

### Data

We conducted a face-to-face survey in 2022 in the Jaffna district of Northern Sri Lanka. The sample includes 628 Tamil civilians aged 18 or older, drawn using a multistage stratified random sampling method across 32 Grama Niladhari (GN) divisions. Respondents answered questions about 35 types of war-related experiences and completed validated instruments measuring PTSD and PTG.

### Research design

Exposure to violence was disaggregated by perpetrator type. For each war-related event, respondents indicated the intensity of exposure (0 to 3 scale) and the identity of the perpetrator (Sinhalese vs. Tamil). These responses were combined into two continuous indices, exposure to intergroup violence and exposure to intragroup violence. PTSD was measured with the 17-item PCL-C (DSM-IV civilian version), asking respondents how much they had been bothered in the past month by each symptom (Cronbach’s α=0.93). PTG was measured with the 10-item Posttraumatic Growth Inventory - Short Form (PTGI-SF), which asks respondents the extent to which they experienced positive psychological changes as a result of the war (Cronbach’s α=0.95).

### Research ethics

The study was approved by the New York University Abu Dhabi IRB (HRPP-2021-191, 2022 January 25). All participants provided informed consent and could skip any question or withdraw at any time without penalty. Enumerators were trained to recognize distress and refer respondents to local counseling and mental health services. To enhance privacy, the survey was self administered on an iPad, and enumerators could not view responses. Research ethics and survey procedures, including survey question wording and order, appear in Appendix S2. Because participation was voluntary and sensitive items could be declined, nonresponse by highly vulnerable individuals likely means our reported mental health outcomes are particularly conservative, which we note when interpreting the results.

### Estimation technique

We estimate the association between wartime violence and mental health outcomes using ordinary least squares (OLS) regression analysis. The models include individual-level controls (age, gender, and education), prewar conditions, and fixed effects for GN to account for unobserved local characteristics. We did not conduct an ex ante power analysis, since the study was a representative survey with sample size set by field feasibility. Ex post, with n=628, α=0.05, and power =0.80, the minimum detectable correlation is r=0.112, while the observed partial correlations for intergroup and intragroup exposure are r=0.162 and r=0.146, respectively, indicating sufficient power.

## Supplementary Material

pgag058_Supplementary_Data

## Data Availability

The code and data necessary to reproduce the results reported in this article are publicly available on Harvard Dataverse at: https://doi.org/10.7910/DVN/VQEFHH
