# Supplementary material for: Mental health impact of intragroup vs. intergroup wartime violence
Source: PNAS Nexus. 2026 Mar 31;5(3):pgag058. doi: 10.1093/pnasnexus/pgag058 (PMC13036719; doi:10.1093/pnasnexus/pgag058)
Supplement: pgag058_Supplementary_Data [file pgag058_supplementary_data.pdf]

Supplementary Information for

Mental Health Impact of Intragroup vs Intergroup Wartime Violence

Table of Contents

Contents

S1 The Effects of Dual Exposure **2**

S2 Research Ethics and Procedures **3**

S2.1 Research Ethics 3

S2.2 Informed Consent and the Option to Stop at Any Time 3

S2.3 Enumerator Training, Referral Procedures, and PI Oversight 3

S2.4 Privacy, Self-Administration, and Oversight 3

S2.5 Survey Question Order 4

S2.6 Survey Question Wording 6

S1 The Effects of Dual Exposure

In this Appendix, we evaluate whether Sinhalese and Tamil, intergroup and intragroup, wartime violence have a joint effect and how it differs from the independent effects presented in the main text. We construct a four-category indicator distinguishing between (i) individuals with low exposure to both types of violence (reference category), (ii) individuals with high exposure only to intergroup violence, (iii) individuals with high exposure only to intragroup violence, and (iv) individuals with high exposure to both.

The correlation between the two exposure measures is modest ($r = 0.15$), and only about 10.6% of respondents fall above the mean on both simultaneously, so the dual-exposure group is relatively small. This still allows us to assess whether concurrent exposure to high levels of intragroup (Tamil) and intergroup (Sinhalese) violence is associated with distinctive postwar outcomes relative to those exposed primarily to only one type.

For PTSD, respondents with high exposure to either Tamil (intragroup) or Sinhalese (intergroup) violence exhibit significantly higher symptom levels than those with low exposure to both. In our preferred specification with GN fixed effects and all controls, PTSD scores are higher by about 5.8 points (standard error 1.7, $p < 0.01$) among those exposed only to Sinhalese violence, by 10.2 points (1.9, $p < 0.01$) among those exposed only to Tamil violence, and by 3.9 points (2.3, $p < 0.10$) among those exposed to both, relative to the low-exposure group. The largest increase is observed for Tamil-only exposure, consistent with intragroup exposure being more strongly associated with PTSD than comparable intergroup exposure. Dual exposure is associated with significantly higher PTSD, but the coefficient is smaller than the Tamil-only effect, suggesting that combined exposure does not amplify PTSD beyond additive effects. The comparatively less severe PTSD among the dual-exposure group is consistent with the idea that the presence of intergroup violence, even alongside intragroup exposure, can trigger blame shifting toward the outgroup.

For PTG, effects are strongest among those primarily exposed to Sinhalese, intergroup, violence. In the same specification, PTG scores are higher by about 10.5 points (1.7, $p < 0.01$) for respondents exposed only to Sinhalese violence, by 4.0 points (1.9, $p < 0.05$) for those exposed only to Tamil violence, and by 8.9 points (2.3, $p < 0.01$) for those exposed to both, relative to the low-exposure group. This pattern matches the evidence in Table 1, where posttraumatic growth arises mainly from intergroup rather than intragroup exposure. Among respondents with dual exposure, the intergroup component appears to dominate, and PTG emerges despite concurrent intragroup violence.

In sum, this analysis shows that intragroup violence is more damaging for PTSD than intergroup violence, whereas intergroup violence is the primary driver of PTG, with only modest PTG associated with intragroup exposure. When individuals experience both forms of violence, their PTSD and PTG outcomes more closely resemble those of respondents primarily exposed to intergroup violence.

S2 Research Ethics and Procedures

S2.1 Research Ethics

This study was reviewed and approved by the New York University Abu Dhabi Institutional Review Board (IRB #HRPP-2021-191, approved January 25, 2022). All research activities adhered to NYUAD's standards for human subjects research and complied with international norms for research ethics. Below, we summarize the main safeguards implemented during the survey.

S2.2 Informed Consent and the Option to Stop at Any Time

Before beginning the survey, participants were informed that some questions could be sensitive. The consent form explained that the interview would cover personal experiences, feelings, and perceptions related to political violence, as well as emotions, trust, and social support. The form stated: *"The interview might lead to discussions about sensitive and potentially distressing experiences related to personal encounters with violence. If, at any point, you feel distressed or uncomfortable, please let us know."* Respondents could decline participation altogether. Those who began the survey retained the right to skip any question or withdraw at any time without penalty.

S2.3 Enumerator Training, Referral Procedures, and PI Oversight

Enumerators received training to recognize signs of distress and to provide referral information for local counseling and mental health services. If a respondent showed visible distress, the enumerator would gently offer to pause or stop the session and provide information on available resources. Enumerators were instructed to monitor and report any distress incidents in the field. One of the project PIs is a psychologist, and she designed and delivered this training to ensure that enumerators could identify distress promptly and initiate culturally appropriate referral procedures. No distress events were recorded, likely because individuals anticipating distress chose not to participate.

S2.4 Privacy, Self-Administration, and Oversight

The survey was self-administered on an iPad to enhance privacy. Enumerators ensured confidentiality by seating the respondent and enumerator alone, face to face, with no one else nearby. This private setup reduced the risk of psychological distress that can arise when sharing sensitive experiences in public or when others might overhear. The enumerator introduced the study, then handed the iPad to the respondent to read the consent form while summarizing key points to confirm understanding. After consent, the respondent completed the survey privately, and the enumerator could not see the screen. The iPad returned to the

enumerator if an attention check failed, prompting the respondent to hand the device back for a reminder to read carefully.

S2.5 Survey Question Order

- **Enumerator Information**
  - Enumerator Name
- **Location Details**
  - Location of the survey (DSD and GN)
- **Literacy Check**
  - Literacy question
  - Literacy task
  - If failed the second attempt: transfer device to enumerator
- **Informed Consent Form**
- **Attention Check**
  - Attention check question
  - If failed: warning
  - Second attempt: attention check question
  - If failed the second attempt: transfer device to enumerator
- **Demographics**
  - **Age**
    - Respondent, Father, Mother
  - **Education**
    - Respondent, Father, Mother
  - **Attention Check**
    - Attention check question
    - If failed: warning
    - Second attempt: attention check question
    - If failed the second attempt: transfer device to enumerator
- **Socio-economic status and residence**

  - **Socio-economic Status**
    * Income, Material well-being, Items in the household
  - **Household Information**
    * Place of residence, Migration History

- **Social and political preferences 1**
  - **Political Engagement**
    * Voting behavior in the last elections
    * Voting behavior of the respondent in 1982
    * Voting behavior of the respondents' parents in 1982
    * Voting behavior of the respondent in 1988
    * Voting behavior of the respondents' parents in 1988
  - **Social preferences**
    * Dictator Game
    * Prejudice
    * Reconciliation
    * Participation in social groups
  - **Attention Check**
    * Attention check question
    * If failed: warning
    * Second attempt: attention check question
    * If failed the second attempt: transfer device to enumerator

- **Traumatic experiences and mental health outcomes**
  - **Others Exposure to Wartime Violence**
    * Personal, family, community exposure, intensity and frequency, and identity of the perpetrator
  - **Mental Health Outcomes**
    * PTSD scale
    * PTG scale (random order)

- **Social and political preferences 2**
  - **Social preferences**
    * Trust Game

- **End of Survey and Compensation Distribution**

S2.6 Survey Question Wording

This appendix reports the exact wording of all survey questions used to construct the variables included in the empirical analysis. When variables are indices or recoded measures, we list the full set of underlying items and response scales.

Informed Consent

Consent

> In accordance with New York University Abu Dhabi (NYUAD) ethics policy, here is a brief standard description of what the study entails.
>
> *Purpose.* The purpose of this study is to get data on experiences, attitudes, and behaviors of people, and monetary decisions people make.
>
> *Volunteer participation.* Your participation in this study is solely on a voluntary basis. You must be at least 18 years old to participate. You are entirely free to participate or not, and you can withdraw at any time.
>
> *Task.* Your task as a participant is to listen to instructions given, answer questions, for some tasks do some trial questions, and respond to questions related to the trial tasks.
>
> *Benefits.* Upon completion of this survey, you can earn up to Rs. 700. However, the exact amount you receive depends on the combined choices that you make and the partner you are paired with in certain tasks in this survey.
>
> *Risks and inconveniences.* The inconvenience of this study is your time commitment. There are also some sensitive questions in this study, but you are free to leave the study at any point. If you do leave the study, no monetary benefit will be given.
>
> *Confidentiality.* Your answers to this survey will be handled confidentially. Personal identifiers such as name or exact home address will not be asked. One question will ask which GN divisions you have lived in.
>
> *Researchers.* This project is led by Dr. Joan Barceló and Keshana Ratnasingham at New York University Abu Dhabi. This research has been reviewed and approved by the NYUAD Institutional Review Board.

Participants were then asked:

> Do you consent to participating in this study?

Response options: *Yes / No.*

—

Demographic Variables

Age

What is your age?

(Open numeric response.)

Gender

What is your gender?

Response options:

- Male
- Female

—

Education Variables

Respondent Education

What was the highest level of education you have obtained?

Response options:

- None
- Nursery
- Kindergarten
- Primary
- Middle-school
- Upper-school
- Vocational training
- Undergraduate
- Post-graduate

Responses were recoded into an ordered numeric variable.

Father's Education

What was the highest level of education obtained by your father?

Response options identical to respondent education.

Mother's Education

What was the highest level of education obtained by your mother?

Response options identical to respondent education.

—

Prewar Household Characteristics

Family Ownership (Prewar)

Did your parents own the building they lived in or rent it?

Response options:

- Own/Built by them
- Rent
- Free

A binary indicator equals 1 if the family owned or built their dwelling.

Family Wealth (Prewar)

When your parents were young, what was their socioeconomic status?

Response options:

- Extremely poor
- Moderately poor
- Neither poor nor rich (basic necessities met)
- Moderately rich
- Extremely rich

Responses were coded into an ordered numeric variable.

—

Geographic Identifier

GN Division

The Grama Niladhari (GN) divisions where the respondents took the survey. This information was used to construct the GN fixed effects variable.

—

Exposure to Wartime Violence

Screening question and event list

Respondents were first shown the following question:

> During the period of war, from 1983 to 2009, which of the following things did you personally directly experience, see or witness with your own eyes and ears, directed at you, your family, or community? We want to remind you that none of the answers you give in this survey will be tied to you and everything will be kept completely anonymous.

For each item below, respondents indicated whether they had experienced it: *No / Yes*.

1. Shootings (even if no one was hurt)
2. Attacks with a weapon (even if no one was hurt)
3. Someone shot bullets directly at you or your home (even if no one was hurt)
4. Suicide bombardment (even if no one was hurt)
5. Artillery bombardment (even if no one was hurt)
6. Bombing from airplanes or missiles (even if no one was hurt)
7. You being seriously wounded
8. Your own family or friends being seriously wounded
9. Other people, not family or friends, being seriously wounded
10. You becoming sexually assaulted
11. Your family or friends being sexually assaulted
12. Other persons, not family or friends, being sexually assaulted
13. Family members or friends being killed
14. Other persons, not family or friends, being killed
15. Family members or friends missing and never seen again
16. Other persons, not family or friends, missing and never seen again
17. The destruction of your belongings/property
18. The destruction of your family's or friends' belongings/property
19. The destruction of other people's, not family or friends' belongings/property
20. Some people tried to abduct you, but you avoided them
21. You were tied up or locked up
22. Your family or friends were tied up or locked up

23. Other people, not your family or friends, were tied up or locked up
24. You had to do forced labor
25. Your family or friends had to do forced labor
26. Other people, not your family or friends, had to do forced labor
27. You were forced to leave your home and move to another part of the country
28. Your family or friends were forced to leave their home and move to another part of the country
29. Other people, not your family or friends, were forced to leave their home and move to another part of the country
30. You had to leave the country and live in a country abroad as a refugee or migrant
31. Your family or friends had to leave the country and live in a country abroad as a refugee or migrant
32. Other people, not your family or friends, had to leave the country and live in a country abroad as a refugee or migrant
33. Your home was raided when you were not there
34. Your family's or friends' home were raided when they were not there
35. Other people's home, not your family's or friends' home, were raided when they were not there

Follow-up questions for endorsed events

For each event that a respondent answered *Yes* to, the survey displayed two follow-up questions: one about the intensity or scope of the experience, and one about the perpetrators.

Intensity / scope questions Depending on the event, the follow-up intensity question took one of the following forms:

- **Frequency of experience** (for most direct-exposure events):
  "In the previous question, you indicated that you experienced [EVENT TEXT] during the period of the war. How often did you experience this?"
  Response options:
  - 1 time only
  - Between 2 and 5 times
  - Between 6 and 9 times
  - 10 or more times
- **Number of persons affected** (for wounding, sexual assault, killings, disappearances):
  "In the previous question, you indicated that you experienced [EVENT TEXT] during the period of the war. How many people did you experience this happen to?"
  (or, when applicable, "How many family members did you experience this happen to?"

/ “How many friends did you experience this happen to?” / “How many people, not including your family and friends, did you experience this happen to?”)
Response options:
  - 1 person only
  - Between 2 and 5 persons
  - Between 6 and 9 persons
  - 10 or more persons

- **Number of properties affected** (for destruction of property):
  “In the previous question, you indicated that you experienced [EVENT TEXT] during the period of the war. How many properties did you experience being destroyed during the war?”
  Response options:
  - 1 property only
  - Between 2 and 5 properties
  - Between 6 and 9 properties
  - 10 or more properties
- **Duration of displacement** (for displacement and refuge abroad):
  “In the previous question, you indicated that you experienced [EVENT TEXT] during the period of the war. For how long did you experience this?”
  Response options:
  - 1 month or less (or: 1 month only)
  - Between 2 and 12 months
  - Between 1 and 3 years
  - Over 3 years
- **Alternative frequency scale** (for some events involving others' property):
  “In the previous question, you indicated that you experienced [EVENT TEXT] during the period of the war. How often did you experience this?”
  Response options:
  - Only once
  - A few times
  - Sometimes
  - Often

Perpetrator identity questions For every endorsed event, a second follow-up question captured perpetrator identity. The wording depended on whether the event involved something *done to* the respondent/others or something the respondent/others were *forced to do*, but the response scale was identical.

For victimization-type events (e.g., bombardment, wounding, killings, disappearances, property destruction), respondents were asked:

And who were the perpetrators of this?

For events where respondents or others were forced to act (e.g., forced labor, being made to beat or kill others, being forced to steal or destroy property, or forced displacement), the question was phrased as:

And who made you do this?

or, when referring to others,

And who made them do this?

In all cases, the response options were:

- People not from your own ethnic group (non-Tamils) only
- Mostly people not from your own ethnic group (non-Tamils), although sometimes people from your own ethnic group (Tamils)
- Both people from your own ethnic group (Tamils) and not from your ethnic group (non-Tamils), to the same extent
- Mostly people from your own ethnic group (Tamils), although sometimes people not from your own ethnic group (non-Tamils)
- People from your own ethnic group (Tamils) only

—

Psychological Outcome Measures

PTSD Symptoms

PTSD symptoms were measured using a 17-item scale. Respondents were instructed as follows:

> Below is a list of problems and complaints that people sometimes have in response to stressful life experiences. Please read each one carefully, indicate how much you have been bothered by that problem in the last month.

Respondents rated each of the following items:

1) Repeated, disturbing memories, thoughts, or images of a stressful experience from the past?
2) Repeated, disturbing dreams of a stressful experience from the past?
3) Suddenly acting or feeling as if a stressful experience were happening again (as if you were reliving it)?
4) Feeling very upset when something reminded you of a stressful experience from the past?

5) Having physical reactions (e.g., heart pounding, trouble breathing, or sweating) when something reminded you of a stressful experience from the past?
6) Avoid thinking about or talking about a stressful experience from the past or avoid having feelings related to it?
7) Avoid activities or situations because they remind you of a stressful experience from the past?
8) Trouble remembering important parts of a stressful experience from the past?
9) Loss of interest in things that you used to enjoy?
10) Feeling distant or cut off from other people?
11) Feeling emotionally numb or being unable to have loving feelings for those close to you?
12) Feeling as if your future will somehow be cut short?
13) Trouble falling or staying asleep?
14) Feeling irritable or having angry outbursts?
15) Having difficulty concentrating?
16) Being "super alert" or watchful on guard?
17) Feeling jumpy or easily startled?

Each item was rated on a five-point scale:

- Not at all
- A little bit
- Moderately
- Quite a bit
- Extremely

The PTSD outcome variable is constructed as the sum of responses across all 17 items.

Post-Traumatic Growth (PTG) Index

Post-traumatic growth was measured using a 10-item scale. Respondents were instructed as follows:

> People sometimes experience changes in response to life experiences. Please read each one carefully, indicate how much you have experienced the following changes as a result of your direct or indirect experiences with the Sri Lankan civil conflict.

Respondents rated the following items:

1. I changed my priorities about what is important in life.
2. I have a greater appreciation for the value of my own life.
3. I am able to do better things with my life.
4. I have a better understanding of spiritual matters.
5. I have a greater sense of closeness with others.
6. I established a new path for my life.

7. I know better that I can handle difficulties.
8. I have a stronger religious faith.
9. I discovered that I'm stronger than I thought I was.
10. I learned a great deal about how wonderful people are.

Each item was rated on a six-point scale:

- I did not experience this change as a result of the war
- I experienced this change to a very small degree as a result of the war
- I experienced this change to a small degree as a result of the war
- I experienced this change to a moderate degree as a result of the war
- I experienced this change to a great degree as a result of the war
- I experienced this change to a very great degree as a result of the war

The PTG outcome is the sum of responses across all 10 items.
